# CREDO: a structural interactomics database for drug discovery

**DOI:** 10.1093/database/bat049

**Published:** 2013-07-18

**Authors:** Adrian M. Schreyer, Tom L. Blundell

**Affiliations:** Department of Biochemistry, University of Cambridge, 80 Tennis Court Road, CB2 1GA Cambridge, UK

## Abstract

CREDO is a unique relational database storing all pairwise atomic interactions of inter- as well as intra-molecular contacts between small molecules and macromolecules found in experimentally determined structures from the Protein Data Bank. These interactions are integrated with further chemical and biological data. The database implements useful data structures and algorithms such as cheminformatics routines to create a comprehensive analysis platform for drug discovery. The database can be accessed through a web-based interface, downloads of data sets and web services at http://www-cryst.bioc.cam.ac.uk/credo.

**Database URL:** http://www-cryst.bioc.cam.ac.uk/credo

## Background

The analysis of the 3D structures of molecules and their interactions in macromolecular complexes has a long history in drug discovery, starting with angiotensin-converting enzyme (1) and renin (2, 3) in the 1980s, and peaking with the successful design of HIV protease inhibitors (4–6) once the structure of the viral enzyme was determined. Since then, a plethora of protein-ligand interaction databases has been developed not only to store the interaction data but also to enable scientists to mine it efficiently (7–9). Moreover, the importance of storing and analysing structures and their interactions has increased in recent years with the ever growing number of publicly available structures in the Protein Data Bank (PDB) (10) and the advent of high-throughput fragment-based drug design (11–13). The latest developments in drug discovery however started to blur the traditional boundaries between the molecules that are involved. The door to a new range of possible drug targets has been opened, for example, with the discovery of small molecules that can bind to shallower surfaces and disrupt interactions between proteins (14, 15). The identification of putative protein–protein interaction (PPI) inhibitors however requires the analysis not only of protein-small molecule but also of PPIs; hence, the existing protein–ligand-only resources will reach their limits quickly. Then, there are the more unusual drug-target interactions that are normally not in the spotlight of drug discovery campaigns but have been commercialized successfully in the past. Small molecules that can intercalate into nucleic acids have been approved for cancer therapy in the past and can be frequently found bound to their targets in the PDB. Interactions between ligands that lead to enzyme inhibition, for example synthetic small molecules that interact with an enzyme’s cofactor, are also a rare but viable strategy for drug discovery and a few drugs combating parasites exploit this mechanism. All these approaches have in common that they transcend the methods a resource focussing on only one kind of structural interaction can offer. Scientists pursuing PPI inhibitors for example raise questions that cannot be answered by traditional resources. These include the following: How do we define and predict hotspots that contribute much of the free energy of a PPI? Which classes of PPIs involve a continuous epitope of one partner and a well-defined groove or series of specific small pockets? What can we learn from PPIs about small molecule binding to the same interface?

We currently do not know of any resources that contain the interactions between all entities in macromolecular complexes on the atomic level—not to mention intramolecular ones. Here, we present a new version of CREDO (7) created to fill this gap. Features from the previous iteration have either been retained or extended, for example complete sequence-to-structure mapping and all protein–ligand interaction features. A range of new features has been implemented, including binding-site similarity searching and protein secondary structure fragments. CREDO also includes information previously available in other databases developed in the Blundell group that store only specific structural interactions: protein–ligand in the old CREDO database (7), protein–protein in PICCOLO (16) and protein–nucleic acid in BIPA (17). The most commonly used features are described later in the text.

## CREDO Web Interface and Web Services

**Figure 1.**
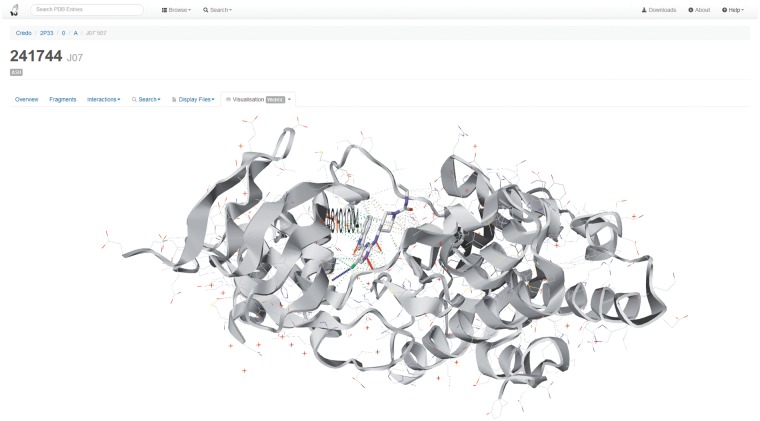
Screenshot of the CREDO website displaying the WebGL-based visualization of the protein–ligand complex [aminopyrimidine inhibitor of c-Jun N-Terminal Kinase (JNK)] found in PDB entry 2P33. The rendering also includes the interactions for ligand J07 as well as a highlighting (residue side chain carbons in pale red) of a polymorphism from dbSNP that can be linked to a binding site-lining residue.

A website has been created to make the CREDO data publicly available in three different forms. The web interface itself allows browsing of the most important entities (PDB entries, ligands, binding sites, etc.) in CREDO, and it also makes commonly used search methods available. The data stored in the database are shown for each entity and augmented with additional external information where possible, e.g. all the assays from ChEMBL a chemical component is part of, are retrieved asynchronously through the ChEMBL web service. Relationships between entities are implemented through internal links visible on each entity page or list. Chemical components for example are linked to their ligands for which the interactions can be seen or even whole protein–ligand complexes can be visualized including the interactions. The visualization of 3D small molecule conformations and biological assemblies has been implemented using a modified version of GLmol (http://webglmol.sourceforge.jp), which itself uses WebGL, a pure JavaScript API supported by most modern browsers to render 2D and 3D graphics (http://www.khronos.org/webgl). A screenshot of this visualization can be seen in Figure 1 for ligand J07 in complex with JNK3 (PDB: 2P33). Moreover, the website can be accessed more programmatically through its RESTful web service—most resources (URLs) can return data sets in JSON format if the request accepts JSON (header contains accept: application/json). All chemical components containing *4-(3-pyridyl*)*pyrimidine* for example can be retrieved with a GET request on the following resource:/credo/chemcomps/?substruct=c1cc(cnc1)c2ccncn2. A description of the available resources and a few examples can be seen in the help section of the website. Finally, the CREDO website also lists the various data sets that have been requested and are available for download. The kinase data set for example contains the interactions between ligands and protein kinase chains that are identified with the help of the regularly updated UniProt human and mouse kinase collection (http://www.uniprot.org/docs/pkinfam). The links to the CREDO website in the following sections also serve as examples of how the database can be accessed.

## The CREDO Database

The CREDO database contains 86 903 PDB entries that define 128 776 biological assemblies having almost 1.2 billion intra- and intermolecular contacts as of release 2013.2.1. Only 96 entries had to be discarded because they contained ligand with insertion codes, aromatic rings split between residues or generally a PDB syntax that was not compatible with the CREDO database schema. For example, in PDB entry 1B01, two observed orientations (chains E and F) of the DNA part were represented as two different alternate location groups, which causes almost super-imposed residues to have different identifiers in addition to completely different chemical component HET-IDs. Polysaccharides are a distinct entity in CREDO as well, although currently there are only a handful of structures (18) that actually contain polymers of this type.

### Stable identifiers

The new CREDO database also introduces stable identifiers for most entities. These identifiers were introduced for several reasons: the sequential integers used as primary keys are valid only for a single database version and hence not stable. Consequently, they cannot be used for external applications such as cross-references to other databases. Stable identifiers in CREDO are implemented as *path trees*, a native data type in PostgreSQL and designed to resemble closely PyMOL selection macros that users of the CREDO database are likely to be familiar with. The stable identifier of ligand J07 in PDB entry 2P33 for example is 2P33/0/A/J07`507, which corresponds to PDB code/biological assembly/chain/residue name`residue number. As can be seen from the example, they only depend on PDB identifiers, meaning they are simple to maintain and, more importantly, they can be deduced by the user. In addition, the true strength of the path tree data type is its powerful regular-expressions-like query syntax in combination with GiST index support. A GiST or generalized search tree index is a data structure supported by PostgreSQL to create custom indexing schemes that are normally not available (http://gist.cs.berkeley.edu). Using ligand path trees as an example, a query for all ligands in PDB entry 1OPJ with the ‘descendant’ operator (<@ ‘1OPJ’) is executed in 10 ms; a query for all Imatinib ligands with the ‘match’ operator (∼ ‘*/STI*’) returns the result in < 100 ms (644 245 ligands are currently in CREDO). These path trees and their expressions can also be used on the CREDO website directly to display data; the resource/ligands/?pmatch=*/STI* for example shows a list of all Imatinib ligands currently in CREDO, whereas/ligands/1IEP/1/B/STI`202 will return the overview page for the corresponding ligand from PDB entry 1IEP. The stable identifiers are also used in database exports that are publicly available as users need to be able to link the downloaded data to their own data sets.

### Structural interactions

**Figure 2.**
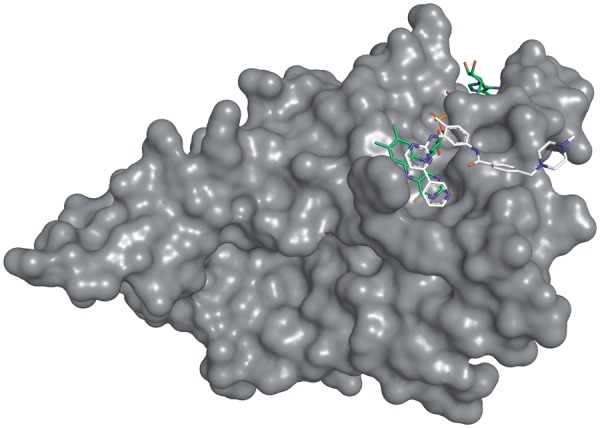
Complex of Imatinib with human NQO2 at 1.75 Å resolution (PDB: 3FW1). Imatinib is displayed with white, FAD with green carbon atoms. Only PDB chain A is shown for clarity.

The new CREDO database now contains the interactions between all *surface-exposed* residues in biological assemblies and classifies them based on the type of entities involved. Surface-exposure is determined for each entity (chain, ligand) and not for the whole assembly. OpenEye’s Spicoli TK is used to generate the grid-based molecular surface for each molecule and to identify which of its atoms are at least partially exposed. Pairwise contacts between all atoms of a structure are stored as Structural Interaction Fingerprints (SIFts) (18–20) with five distance-based and eight feature contact types that have distance, atom type and possible geometry constraints. A unique feature of CREDO is the integration of halogen bonds (21–23) and carbonyl interactions (24) into the SIFts. A clear terminology is used to distinguish structural interactions between entities: interactions between proteins and ligands are called *binding sites*, *interfaces* between polypeptide chains and *grooves* between a polypeptide and an oligonucleotide. Besides these more familiar molecular interactions, there are also lesser-known types that can be of significant interest to the drug discovery community such as ligand–ligand or ligand–nucleic acid interactions. Interactions between small molecules and cofactors that are required to catalyze enzymatic reactions are already known as inhibitory mechanism to target pathogens. Anti-tubercular drugs targeting the long-chain enoyl-acyl carrier protein reductase (*InhA*) work by binding covalently to nicotinamide adenine dinucleotide hydride, thereby inhibiting the synthesis of mycolic acid, which is required for the mycobacterial cell wall. Inhibition through ligand–ligand interactions is not limited to pathways in pathogens however; Winger *et al.* (25) demonstrated that the anticancer drug Imatinib not only inhibits several kinases but also quinone reductase 2 (NQO2) at 80 nM by interacting with FAD. The crystal structure of NQO2 in complex with FAD and Imatinib is shown in Figure 2.

### Interactions between aromatic rings and atom–aromatic interactions

**Figure 3.**
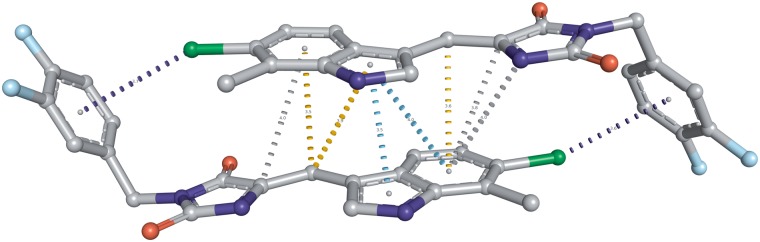
Interactions between aromatic rings as well as atoms and aromatic rings in the dimeric small molecule complex in the p53 pocket of MDM2 (PDB: 3VBG). The inhibitor complex leads to the dimerization of MDM2 and MDMX proteins, eventually causing cell cycle arrest and apoptosis. Aromatic–ring interactions are displayed using a cyan dashed line, halogen-pi interactions are shown as dark blue dashed line, carbon-pi in orange and undefined atom-ring interactions simply in grey. Only interactions within 4.0 Å are shown.

Interactions between aromatics rings as well as the interactions between atoms and aromatic rings are explicitly recorded and classified in CREDO. The geometries of all aromatic ring interactions are calculated inside the database using a linear algebra extension called *pgeigen* (http://hg.adrianschreyer.eu/pgeigen) and classified into nine groups according to Chakrabarti and Bhattacharyya (26). Atoms in close proximity to aromatic rings are also stored in CREDO as atom–ring interactions along with their distance to the ring’s centroid and the θ angle between the normal of the aromatic ring and the vector between the centroid and the close atom. Only interactions having a θ angle ≤30.0° are included to reduce the number of false positives. Atom–ring interactions are labelled if specific atom type constraints are satisfied: *donor-pi* if the atom is a hydrogen bond donor, *carbon-pi* if the atom element is carbon and a weak hydrogen bond donor, *cation-pi* for positively ionisable atoms and *halogen-pi* in case of halogen bond donors. A nice example showing the relevance of these interactions in drug discovery is the structure of human MDM2 in complex with a dimer-inducing inhibitor (PDB: 3VBG). The ligands form a dimer in the p53-binding pocket of the protein, which leads to the dimerization of the protein and ultimately to cell cycle arrest and apoptosis (27). The ring–ring and atom–aromatic ring interactions (using a 4.0 Å cut-off) that are involved in forming the dimer can be seen in Figure 3.

### Quality assessment of macromolecular structures

Structures determined by protein X-ray crystallography or Nuclear Magnetic Resonance have unavoidable imprecision. Many analyses and applications in drug discovery on the other hand require high-quality structures that fulfil certain quality criteria such as having structure factors (resolution, 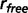, etc.) within an acceptable range or structural characteristics have to be present, e.g. all atoms in a ligand-binding site are observed. Warren *et al.* (28) reviewed methods for the assessment of protein–ligand structure quality and proposed a new set of criteria that can be used to identify high-quality structures. Several of these measures that can be used to assess structure quality and do not rely on electron density maps have now been introduced into the CREDO database since the initial publication. An important measure that is widely used nowadays in the drug discovery community is the *Diffraction-Component Precision Index* (DPI) (29, 30). The DPI is a *global* measure of atom coordinate uncertainty in structured determined X-ray crystallography. It has been calculated using Goto’s formula, which calculates the coordinate error in the distance and not a particular axis (31), for all PDB structures in CREDO where the necessary structure factors were available (67 273). In addition, Blow’s rearranged formula (Goto’s version) has been used to calculate the theoretically smallest atom coordinate imprecision possible at the nominal resolution of a structure.

A few additional quality measures have been added to database entities in the form of Boolean flags, one of them, for example, is set to indicate the absence of atoms in the PDB residues that are stored in CREDO. Proteins often contain disordered regions such as loops and other flexible regions that are more difficult to observe in the electron density. More important in a drug discovery context is to know that the side chains of residues lining the binding sites and those situated in protein–protein interfaces do not have any missing atoms, which can easily be overlooked. In all, 1194 of the 11,577 binding sites of drug-like ligands in CREDO have missing atoms for example as do 49,983 of the 266,062 interfaces with at least 10 interacting residues on each side. As a result, creating a high-quality subset from the CREDO database is straightforward with the availability of structure factors, qualitative measures such as the DPI and the Boolean flags.

### Protein-ligand interactions

Inter1actions between proteins and ligands remain an important aspect of the CREDO database. Therefore, all the features of the previous version of CREDO concerning protein-ligand interactions have either been retained or extended. Ligand efficiencies from the ChEMBL database (32), for example, have been added to annotate protein-ligand complexes in CREDO. As a result, 6505 ligands could be successfully linked to 6848 unique activity values. The total number of ligand-activity pairs was 28,943 because in some cases, multiple PDB entries could be matched with activity data and quaternary ligands that are not in the same crystallographic asymmetric unit could be linked as well.

**Figure 4.**
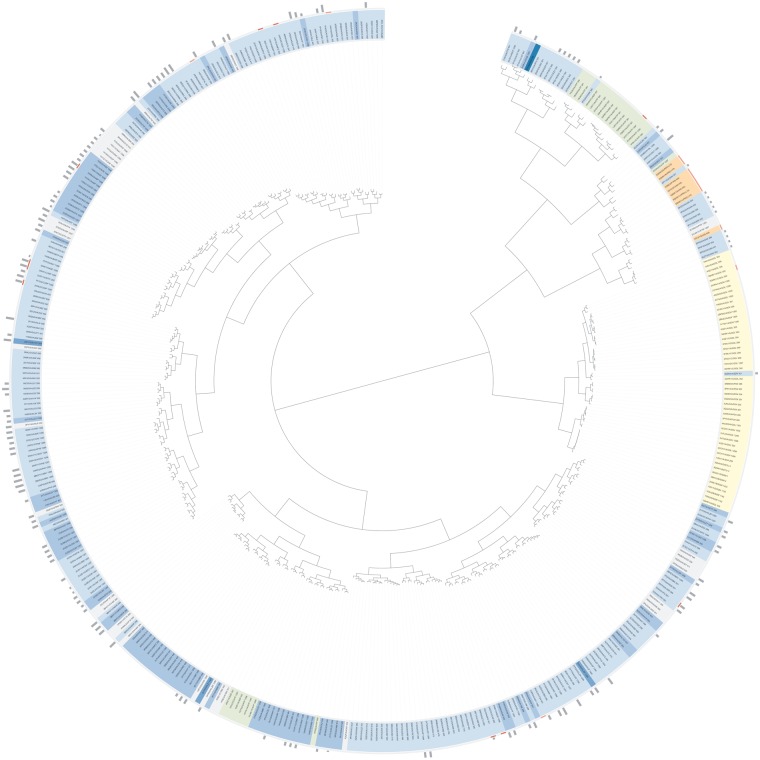
SIFt clustering for all ligands binding to UniProt entry P24941, Cyclin-dependent kinase 2.

Ligands in CREDO were also clustered using their SIFts. These clusters can be analysed in various ways depending on the objective; common interaction patterns can quickly be identified, e.g. which key interactions distinguish a ligand with high affinity from others. In addition, they can be used to analyse the impact of binding site mutations, in the context of drug resistance for example. Here, SIFts are simply 1D fingerprints that encode the specific interactions that a ligand has with binding site residues that are *aligned* to a coordinate system. The UniProt sequences of the protein chains were used to align the protein–ligand interactions and stacked into a matrix where each block of positions belongs to the same residue. These SIFts matrices were eventually used to perform a hierarchical clustering, and the results stored in the database with additional calculated properties for the leaf nodes such as topological as well USRCAT (33) and ROCS shape similarity (34). The node properties make it possible, for example, to query the database for neighbours, i.e. ligands that have the most similar interactions with a protein to a given ligand of interest. This feature is also implemented in the web interface under the ‘search’ tab. It is also straightforward to look for cases where ligands have similar interactions but large topological dissimilarity. The ligands in the pair formed by PP0 from PDB entry 3IW6 and N3F (PDB: 3QUD) in the cluster of human p38 MAP Kinase, for example, have a topological Tanimoto similarity of only 0.13. Conversely, one could search for pairs with high degree of 2D similarity but large difference in shape—this would indicate that not all ligand atoms are contributing to the interaction and that the remaining atoms are sticking into the bulk solvent. The UniProt SIFt clusters, as they are called in CREDO, can be visualized dynamically as circular phylogenetic trees through the web interface. The open-source JavaScript library jsPhyloSVG (35) is used to render the clusters as vector graphics (SVG) with highlighting of node and leaf properties. One of the largest clusters, the protein–ligand interactions for cyclin-dependent kinase 2 (UniProt: P24941), can be seen in Figure 4. A high-resolution version is also available in the supporting information.

The ligand (leaf) nodes link directly to the corresponding ligand. The background colour of the ligands reflects the class of the chemical component—the blue shades are used to represent drug-likeness, with light blue for drug-like compounds and dark blue for approved drugs. Green is used for nucleotides (includes ATP), orange for heteropeptides and yellow for solvents. An internal arc chart is used to indicate the type of protein–ligand interaction (blue for drug-target, green for enzyme-substrate/product) if possible and an external arc chart reflects binding site properties, i.e. light red if at least one peptide is modified and red if the binding site contains a mutation. The stacked bars on top of the outermost circle display the best-mapped activity values (pKd) from ChEMBL.

### RECAP fragmentation of chemical components

The RECAP (36) fragmentation of chemical components that was described in the previous publication has been augmented with several new rules to improve the fragmentation of natural products containing phosphate groups for example. Fragments have been mapped back onto ligands and their atoms in the new version to simplify their analysis in the context of structural interactions. The mapping also made it possible to devise a new measure to identify fragments of ligands that have a proportionally higher number of contacts. This measure, called *fragment contact density* (FCD), is simply the ratio between the number of contacts per fragment atom and the number of contacts per ligand atom [Equation (1)].
(1)
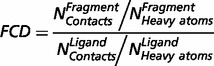



**Figure 5.**
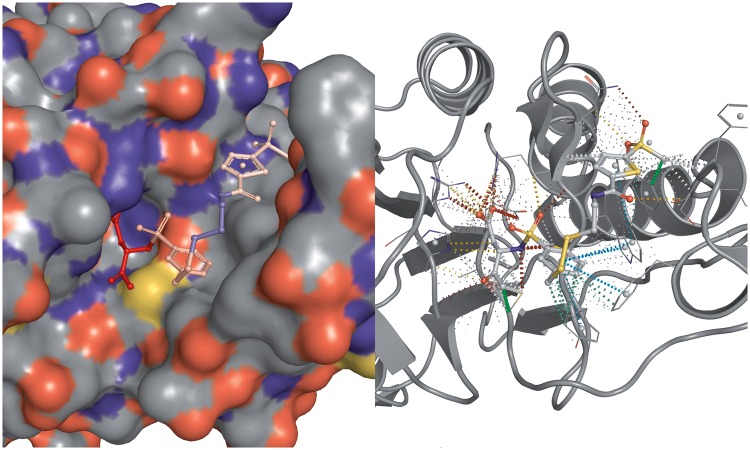
FCD visualized in the crystal structure of cysteine aspartyl protease-3 (caspase-3) in complex with a non-peptidic inhibitor. The image was rendered as grid in PyMOL, atomic positions are identical on both sides. The FCD (from blue to red) is shown for the set of smallest fragments resulting from the fragmentation of this ligand, chemical component 160.

The FCD can also be visualized in PyMOL and an example is shown in Figure 5 for the structure of cysteine aspartyl protease-3 (caspase-3) in complex with a non-peptidic inhibitor (PDB: 1NMQ).

### Cheminformatics

Finding the protein–ligand complexes of interest often requires the use of chemical query tools (e.g. database cartridges) to search for chemical patterns or chemically similar compounds. All the cheminformatics features of the previous CREDO version have been either retained or extended for that purpose. Two PostgreSQL extensions have been installed to augment the database server with a comprehensive set of cheminformatics functions and data types: the extension from the open source RDKit (37) and *pgopeneye*, an in-house extension that makes functions from several OpenEye C++ toolkits (OpenEye Scientific Software, Santa Fe, NM. http://www.eyesopen.com) accessible as SQL functions. Both cartridges support a wide range of cheminformatics functions, including substructure and pattern matches as well as topological fingerprints with GiST index support. Additional properties of chemical components have been introduced as well, most notably InChI keys and the *Quantitative Estimate of Drug-likeness* (QED) descriptor (38). The most drug-like chemical components according to QED, for example, in the PDB can be retrieved through the following resource: /chemcomps/?orderby=ChemComp.qed.desc() .nullslast().

The database server also has a native implementation of USRCAT (33), an extension of the Ultrafast Shape Recognition (USR) algorithm (39, 40) that includes pharmacophoric information. All chemical components that have 3D shapes similar to Imatinib (HET-ID: STI), for example, can be seen (or retrieved) on the website at/chemcomps/sim/usr/?het_id=sti&conformer=1&usrcat=t. USRCAT moments have been calculated for all ligands (and their up to 25 modelled conformers) in CREDO with at least seven heavy atoms (currently ∼250 000 conformers). Results are typically returned in <50 ms from the database. It is possible to perform a USRCAT search of ligands or chemical components with an uploaded structure either through a form on the website or more programmatically through a POST request (see API section on the website). Uploading chemical structures is not possible for many researchers though due to IP restrictions; therefore, the same queries can be performed by posting only the calculated USRCAT moments of a molecule. The USRCAT implementation is freely available and can be used to generate these moments internally (moments cannot be used to reverse engineer the original set of coordinates).

**Figure 6.**
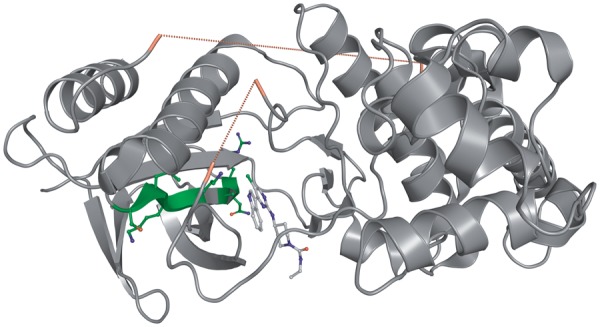
Visualization of missing regions and a secondary structure fragment in PDB entry 2P33. The two regions of missing amino acids inside are displayed with a red dashed line that connects the Cα atoms of the flanking residues. N- and C-terminal missing regions are not shown. The secondary structure fragment 2P33/0/A/PF:13, an extended beta strand, is visualized with amino acid side chains and carbon atoms in green.

### Binding site similarity

The FuzCav algorithm (41) has been implemented natively in the database to enable fast similarity searching of protein–ligand-binding sites. An additional FuzCav fingerprint type has been added that uses representative amino acid side-chain atoms (42). It is also possible to use different similarity metrics to optimise the search results, for example if globally similar hits are desirable or rather those with local similarity. FuzCav fingerprints have been calculated for all protein-binding sites defined by ligands with at least seven heavy atoms that are not clashing with the binding site’s atoms, leading to a total number of 159,676. The database implementation also allows the use of more advanced filters to modify the binding site search. Ligands can be included or excluded based on their properties, interactions and so forth, as can the binding sites, to filter based on UniProt accession or CATH/SCOP structural classifications for instance.

### Variations affecting molecular interactions

Where possible, variations from EnsEMBL Variation (43) are mapped onto peptide residues in CREDO through sequence-to-structure mapping. The EnsEMBL Variation database contains a collection of disease phenotypes that are associated with specific variations through the analysis of internal and external studies. These phenotypes are directly mapped to entities (peptide, chains, etc.) as well as to structural interactions such as binding sites, interfaces and grooves, which means it is possible to retrieve those simply through a keyword search on the phenotype descriptions. In all, 2457 disease phenotypes could be linked to surface-exposed peptide residues in CREDO, 1130 of these could also be directly linked to peptides interacting across protein–protein interfaces, 1012 to protein–ligand-binding sites and 44 to protein–nucleic acid grooves. The binding site of ligand 2OBD/0/A/2OB`486 for example could be linked to EnsEMBL phenotype 5825, high density lipoprotein (*HDL*) *cholesterol* (/bindingsites/?phenotype_id=5825). A closer look at the PDB entry reveals that the polymer is a cholesteryl ester transfer protein whose inhibition is known to raise HDL cholesterol (44). Hence, it is possible to infer that the A373P polymorphism (rs5880), which is in direct contact with a cholesteryl ester, likely suppresses the transfer activity of the protein, thereby leading to elevated HDL levels. The CREDO website allows searching and browsing of phenotypes, and direct links to other entities in CREDO are displayed for each (e.g. target chains, residues, binding sites, etc.).

### Secondary structure fragments

Polypeptide chains have been divided into fragments using secondary structure information from the sequence-to-structure mapping that also contains the relevant data in the form of DSSP predictions. A secondary structure (or protein) fragment in CREDO is simply a sequence of adjacent peptide residues that share the same secondary structure code. The fragment 2P33/0/A/PF:13, for example, is an extended beta strand (E) with the amino acid sequence RNVAIKKLS. The protein fragment entity is deeply connected to the other entities in CREDO, which makes it possible for example to retrieve all the fragments that another similar protein fragment or ligand is interacting with. The CREDO web interface displays the protein fragments that a ligand is in contact with by default. Closely related to secondary structure fragments are regions of missing amino acid residues in polypeptide chains. These missing regions are not immediately obvious to see in molecular modelling software. An example is shown in Figure 6 for PDB entry 2P33 with the missing regions illustrated as red dashed lines that connect the flanking residues.

## References

[1] Ondetti MA, Cushman DW (1982). Enzymes of the renin-angiotensin system and their inhibitors. Ann. Rev. Biochem..

[2] Szelke M, Leckie B, Hallett A (1982). Potent new inhibitors of human renin. Nature.

[3] Blundell T, Sibanda BL, Pearl L (1983). Three-dimensional structure, specificity and catalytic mechanism of renin. Nature.

[4] Miller M, Schneider J, Sathyanarayana BK (1989). Structure of complex of synthetic HIV-1 protease with a substrate-based inhibitor at 2.3 A resolution. Science.

[5] Lapatto R, Blundell T, Hemmings A (1989). X-ray analysis of HIV-1 proteinase at 2.7 A resolution confirms structural homology among retroviral enzymes. Nature.

[6] Wlodawer A (2002). Rational approach to AIDS drug design through structural biology. Ann. Rev. Med..

[7] Schreyer A, Blundell T (2009). CREDO: A Protein-Ligand Interaction Database for Drug Discovery. Chem. Biol. Drug Des..

[8] Weisel M, Bitter HM, Diederich F (2012). PROLIX: rapid mining of protein-ligand interactions in large crystal structure databases. J. Chem. Inf. Model..

[9] Roche O, Kiyama R, Brooks C (2001). Ligand-protein database: linking protein-ligand complex structures to binding data. J. Med. Chem..

[10] Berman HM, Westbrook J, Feng Z (2000). The protein data bank. Nucleic Acids Res..

[11] Rees D, Congreve M, Murray C (2004). Fragment-based lead discovery. Nat. Rev. Drug. Discov..

[12] Greer J, Hajduk P A decade of fragment-based drug design: strategic advances and lessons learned. Nat. Rev. Drug Discov..

[13] Blundell TL, Jhoti H, Abell C (2002). High-throughput crystallography for lead discovery in drug design. Nat. Rev. Drug Discov..

[14] Higueruelo AP, Schreyer A (2009). Atomic interactions and profile of small molecules disrupting protei1n-protein interfaces: the TIMBAL database. Chem. Biol. Drug Design.

[15] Winter A, Higueruelo AP, Marsh M (2012). Biophysical and computational fragment-based approaches to targeting protein-protein interactions: applications in structure-guided drug discovery. Q. Rev. Biophys..

[16] Bickerton GR, Higueruelo AP, Blundell TL (2011). Comprehensive, atomic-level characterization of structurally characterized protein-protein interactions: the PICCOLO database. BMC bioinformatics.

[17] Lee S, Blundell TL (2009). BIPA: a database for protein-nucleic acid interaction in 3D structures. Bioinformatics.

[18] Brewerton S (2008). The use of protein-ligand interaction fingerprints in docking. Curr. Opin. in Drug Discovery & development.

[19] Deng Z, Chuaqui C, Singh J (2004). Structural interaction fingerprint (SIFt): a novel method for analyzing three-dimensional protein-ligand binding interactions. J. Med. Chem..

[20] Chuaqui C, Deng Z, Singh J Structural interaction fingerprint (SIFt): a novel method for analyzing three-dimensional protein-ligand binding interactions. J. Med. Chem..

[21] Voth A, Hays F, Ho P (2007). Directing macromolecular conformation through halogen bonds. Proc. Natl Acad. Sci. USA.

[22] Auffinger P, Hays FA, Westhof E, Ho PS (2004). Halogen bonds in biological molecules. Proc. Natl Acad. Sci. USA.

[23] Bissantz C, Kuhn B, Stahl M (2010). A medicinal chemist’s guide to molecular interactions. Journal of medicinal chemistry.

[24] Allen FH, Baalham CA, Lommerse JPM, Raithby PR (1998). Carbonyl-Carbonyl Interactions can be Competitive with Hydrogen Bonds. Acta Crystallogr. Sec. B.

[25] Winger J, Hantschel O, Furga GS (2009). The structure of the leukemia drug imatinib bound to human quinone reductase 2 (NQO2). BMC Struct. Biol..

[26] Chakrabarti P, Bhattacharyya R (2007). Geometry of nonbonded interactions involving planar groups in proteins. Progr. Biophys. Mol. Biol..

[27] Graves B, Thompson T, Xia M (2012). Activation of the p53 pathway by small-molecule-induced MDM2 and MDMX dimerization. Proc. Natl Acad. Sci. USA.

[28] Warren GL, Do TD, Kelley BP (2012). Essential considerations for using protein-ligand structures in drug discovery. Drug Discov. Today.

[29] Cruickshank DWJ (1999). Remarks about protein structure precision. Acta Crystallogr. Sec D Biol Crystallogr.

[30] Blow DM (2002). Rearrangement of cruickshank’s formulae for the diffraction-component precision index. Acta Crystallogr. Sec D Biol Crystallogr..

[31] Goto J, Kataoka R, Muta H (2008). ASEDock-docking based on alpha spheres and excluded volumes. J. Chem. Inf. Modeling.

[32] Gaulton A, Bellis LJ, Bento AP (2012). ChEMBL: a large-scale bioactivity database for drug discovery. Nucleic Acids Res..

[33] Schreyer AM, Blundell T (2012). USRCAT: real-time ultrafast shape recognition with pharmacophoric constraints. J. Cheminform..

[34] Nicholls A, McGaughey GB, Sheridan RP (2010). Molecular shape and medicinal chemistry: a perspective. J. Med. Chem..

[35] Smits SA, Ouverney CC (2010). jsPhyloSVG: a javascript library for visualizing interactive and vector-based phylogenetic trees on the web. PLoS One.

[36] Lewell XQ, Judd DB, Watson SP (1998). RECAP-Retrosynthetic Combinatorial analysis procedure: a powerful new technique for identifying privileged molecular fragments with useful applications in combinatorial chemistry. J. Chem. Inf. Model..

[37] http://www.rdkit.org.

[38] Bickerton GR, Paolini G V, Besnard J (2012). Quantifying the chemical beauty of drugs. Nat. Chem..

[39] Ballester PJ, Richards WG Ultrafast shape recognition to search compound databases for similar molecular shapes. J. Comput. Chem..

[40] Ballester PJ, Westwood I, Laurieri N (2010). Prospective virtual screening with Ultrafast Shape Recognition: the identification of novel inhibitors of arylamine N-acetyltransferases. J. R. Soc. Interface.

[41] Weill N, Rognan D (2010). Alignment-free ultra-high-throughput comparison of druggable protein−ligand binding sites. J. Chem. Inform. Model..

[42] Tanramluk D, Schreyer A, Pitt WR (2009). On the origins of enzyme inhibitor selectivity and promiscuity: a case study of protein kinase binding to staurosporine. Chem. Biol. Drug Des..

[43] Hubbard TJ, Aken BL, Beal K (2007). Ensembl 2007. Nucleic Acids Res..

[44] Qiu X, Mistry A, Ammirati MJ (2007). Crystal structure of cholesteryl ester transfer protein reveals a long tunnel and four bound lipid molecules. Nat. Struct. Mol. Biol..

